# Coupled agent‐based and hyperelastic modelling of the left ventricle post‐myocardial infarction

**DOI:** 10.1002/cnm.3155

**Published:** 2018-10-22

**Authors:** Xin Zhuan, Xiaoyu Luo, Hao Gao, Ray W. Ogden

**Affiliations:** ^1^ School of Mathematics and Statistics University of Glasgow Glasgow UK

**Keywords:** agent‐based model, fibre orientation, finite element method, left ventricular modelling, myocardial infarction

## Abstract

Understanding the healing and remodelling processes induced by myocardial infarction (MI) of the heart is important, and the mechanical properties of the myocardium post‐MI can be indicative for effective treatments aimed at avoiding eventual heart failure. MI remodelling is a multiscale feedback process between the mechanical loading and cellular adaptation. In this paper, we use an agent‐based model to describe collagen remodelling by fibroblasts regulated by chemical and mechanical cues after acute MI, and upscale into a finite element 3D left ventricular model. We model the dispersed collagen fibre structure using the angular integration method and have incorporated a collagen fibre tension‐compression switch in the left ventricle (LV) model. This enables us to study the scar healing (collagen deposition, degradation, and reorientation) of a rat heart post‐MI. Our results, in terms of collagen accumulation and alignment, compare well with published experimental data. In addition, we show that different shapes of the MI region can affect the collagen remodelling, and in particular, the mechanical cue plays an important role in the healing process.

## 1 INTRODUCTION

Myocardial infarction (MI), commonly known as a heart attack, occurs when blood flow decreases or stops to a part of the heart, causing damage to the heart muscle. The healing process in the heart poses a complex multiscale soft tissue problem, involving cardiac growth and remodelling (G&R). Cellular growth is often considered to be the cause for residual stress.^1^ The myocardial stress and constitutive properties are considered to be the two key factors in myocardial G&R.^2^ There are typically two types of G&R modelling approaches at the continuum level. One is the volumetric growth (the density remains unchanged) following the G&R, and the other is the density growth (volume remains unchanged). In the volumetric approach, G&R can be modelled using the growth tensor first introduced by Rödriguez et al.^3^ For example, Göketepe et al^4^ developed a multiscale framework to study myocardial growth via alignment of myocytes and parallel additional of sarcomere units. Their model was extended to study the mechanical effects on remodelling patterns of the left ventricle (LV) after certain surgical procedures.^5^, ^6^ Kerckhoffs et al^7^ developed a strain‐driven growth law to explain how the grown cardiac myocytes increased the fibre and cross‐fibre strains.^8^ Density growth, on the other hand, focuses on changing the constitutive properties of myocaridum in healthy remote and infarcted cardiac tissues, the latter being characterised with stiffer constitutive properties, simulating the replacement of fibrosis by collagen fibres.^9^, ^10^, ^11^ For example, in a study,^12^ the stiffness in the infarct zone is increased significantly compared with functional myocardium in a patient‐specific infarcted LV model.

There are very few combined volumetric and density growth models for G&R, particularly for the heart. Eriksson et al^13^ developed a framework to study the G&R process of fibre‐reinforced arteries, while the evolution of constitutive properties was simulated via a density‐and‐volumetric growth approach for a mixture of constituents. However, the remodelling of the fibre structure was not included. Indeed, it is not clear how the collagen fibre structure is modified during the post‐MI healing process, and how the macromechanical response of the infarcted LV changes post‐MI.

Recent developments in soft tissue modelling in parallel with advances in experimental techniques have enabled us to predict mechanical states at, and obtain images of, the small scales relevant to biology. For example, the structure‐based fibre‐reinforced Holzapfel‐Ogden (HO) model^14^ for myocardium was developed based on simple shear experiments and has catalysed much of the recent activities in soft tissue modelling.

At the cellular level, collagen deposition and remodelling are regulated by fibroblast cell alignment. Environmental cues, such as mechanical and chemical cues, have been shown to influence cell migration and regulate the microfibre structures.^15^ Recently, agent‐based models that account for these effects have been developed and used to study a two‐dimensional (2D) slab model of the myocardium infarction.^16^, ^17^ However, the extension of this approach to a three‐dimensional (3D) LV model has not been reported.

In this paper, we combine an agent‐based approach and a structure‐based fibre‐reinforced constitutive law to study MI healing in a 3D LV model for the first time. To account for the necessarily dispersed fibre structure post‐MI, we modify the original HO constitutive law^14^ by employing a distributed fibre model.^18^ The specific fibre distribution is determined using an agent‐based model similar to that of Fomovsky & Holmes.^16^ We then incorporate the agent‐based model within a finite element (FE) LV model. This new mathematical model is used to simulate the myocardium remodelling in terms of the collagen fibre structure and density. To account for the microfibre structure in the tissue constitutive laws, a commonly used up‐scaling method is based on volumetric averaging.^19^ However, since these microfibres in the soft tissue do not support compression, a switch is required to exclude the compressed fibres. For a dispersed fibre structure, however, such a “switch” is often applied inappropriately due to the volume averaging.^18^ In our model, we describe the fibre structure using angular integration with an analytical expression for the fibre on‐off switch, which is then implemented in the constitutive model.

## 2 METHODOLOGY

Here, we describe the model in detail. For convenience, the list of the variables used in the model is defined in Table 1.

**Table 1:** List of variables

Basis vectors of local coordinates (in current configuration)	$\left\{c_{0},l_{0},n_{0}\right\}\;(\left\{c,l,n\right\})$
Chemokine concentration field	*C*(·)
Right Cauchy‐ Green deformation tensor	**C**
Eulerian strain tensor	**D**
Collagen fibre vector (in current configuration)	**f**_0_(**f**)
Deformation gradient	**F**
*i*th invariant of **C**	*I*_*i*_
Unit outward normal vector of the cell surface	**n**_*c*_
Total collagen fibre number	*N*_tot_
Number of collagen fibres along angle of *θ*	*N*(*θ*)
Rotation tensor	**Q**
Unit vector for the *i*th cue	**v**_*i*_
Distance to the infarction centre	*r*
Position vector (in current configuration)	**X**(**x**)
Centre of the MI	**X**_*C*_
Normal strain	*ε*_*n*_
Tuned resultant cue vector	***ρ***
Delta function	*δ*
Strain energy function	Ψ
Volumetric fraction of collagen fibre (matrix) at time *t*	Φ_cf,*t*_(Φ_m,*t*_)
Cauchy stress	***σ***

Abbreviation: MI, myocardial infarction.

### 2.1 The geometry of the LV model

**Figure 1:**
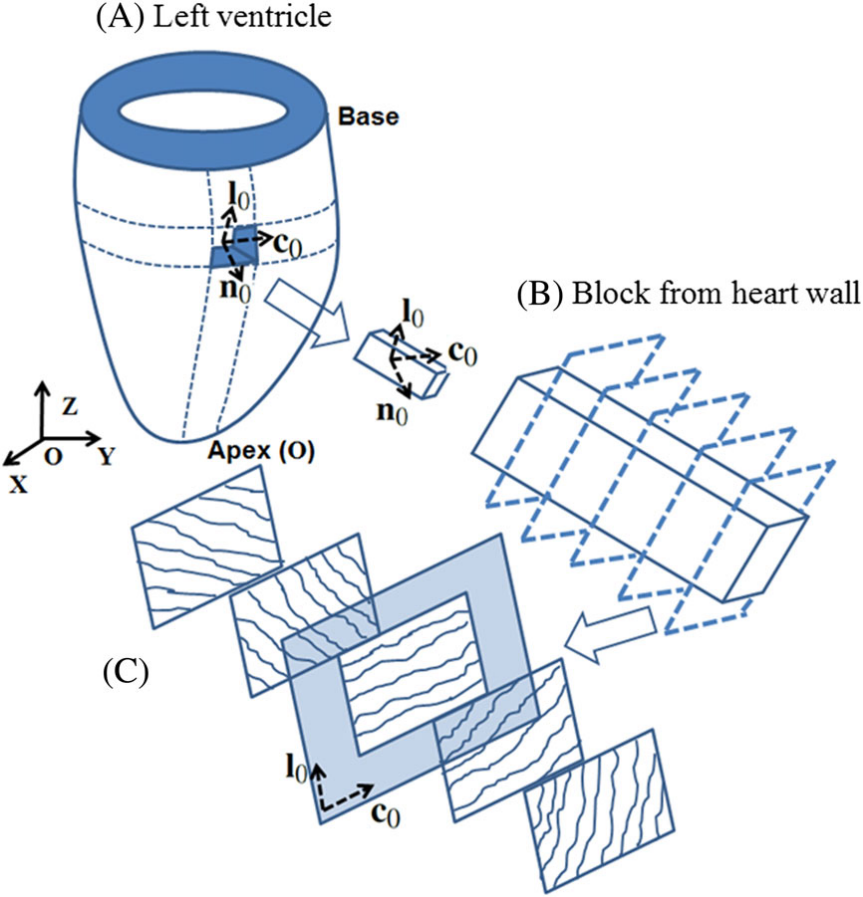
A, The left ventricle (LV) geometry with 28‐mm‐long axis, internal radius of 5 mm and external radius of 10 mm at the base, and a block cut from the LV wall. The ratio between the LV wall thickness and the internal radius is chosen to be 1 following Omens and Fung.^20^ The basis vectors at the reference configuration are (**c**_0_, **l**_0_, **n**_0_) for local coordinates, where **c**_0_, **l**_0_, and **n**_0_ are the local circumferential, longitudinal, and transmural unit vectors. The basis vectors at the reference configuration are (**X**, **Y**, **Z**) for global Cartesian coordinates, with origin **O** at the LV apex. B, The fibre structure through the thickness of the LV wall. C, Five longitudinal–circumferential sections through the wall thickness. Collagen fibres lie in the **c**_0_ − **l**_0_ plane

An idealised half ellipsoid geometry is used to construct a FE model for a rat LV, with 2100 eight‐node hexahedral elements and 2375 nodes (Figure 1). Global Cartesian coordinates (*X*,*Y*,*Z*) are used to describe material points in the undeformed reference configuration, with the corresponding basis vectors denoted {**E**_*X*_,**E**_*Y*_,**E**_*Z*_}.

Myocardium is considered to be a fibre‐reinforced material mostly composed of collagen fibres and myocytes.^14^ A local coordinate system with the circumferential, longitudinal, and transmural basis vectors (**c**_0_, **l**_0_, **n**_0_), is introduced to describe the layered fibre structure within the ventricular wall, as shown in Figure 1. Note that

(1) $$c_{0}=E_{Z}\times n_{0},\;l_{0}=n_{0}\times c_{0}.$$

The myofibre architecture is generally described by a “fibre‐sheet‐normal” system (**f**,**s**,**sn**)^21^ in the current configuration. Here, we assume that the fibre direction **f** always lies in the **c** − **l** plane, the sheet direction is transmural, and the sheet‐normal **sn**  =  **f** × **s**, where **c** and **l** are the current circumferential and longitudinal directions: **c**  =  **Fc**_0_,**l**  =  **Fl**_0_, and **F** is the deformation gradient.

### 2.2 Agent‐based model

#### 2.2.1 Chemokine concentration

Fibroblast cells adjust the microstructure of heart tissue, including the density and orientation of the collagen fibre bundles, which determine the material properties of infarcted tissues. The collagen fibres are organized in a highly layered architecture in healthy tissues. Following an acute MI, sudden changes of the chemokine concentration and mechanical environment in and around the infarct area activate the fibroblasts, which will remodel the micro‐collagen structure of heart tissues by changing the collagen orientations and its volume fraction through deposition and degradation. Subsequently, the material properties and mechanical behaviour are modified at the tissue level. In this study, we extend the 2D agent‐based model developed in Fomovsky and Holmes^16^ to 3D, to describe the collagen remodelling post‐MI. The modified 3D agent‐based model is explained in the coming section.

We assume a spherical MI with its centre at **X**_*c*_. The static chemokine concentration at point **X**, representing the milieu of cytokine and chemokine surroundings, is described by a chemical diffusion equation as

(2) $$D_{c}\nabla^{2}C(X)=\left\{\begin{array}{cc} k_{\text{c,deg}}C(X)-k_{\text{c,gen}}, & X\;\in \Omega_{b}\\ k_{\text{c,deg}}C(X), & X\;\notin \Omega_{b}, \end{array}\right.$$

where Ω_*b*_ is the volume of the infarct region, *D*_*c*_ is the diffusion coefficient, *C* is the chemokine concentration, and *k*_c,gen_, *k*_c,deg_ are the chemokine generation and degradation rates, respectively.

The boundary condition for the chemokine concentration equation is

(3) $$C(X)=0,\;\text{if}\;||X-X_{c}||\to \infty .$$

Experimental data suggest that the infarct‐induced chemical concentration reduces rapidly from the infarct area, and drops to nearly zero away from the infarct centre. Hence, in practice, we choose *C*(**X**)  =  0 when ||**X** − **X**_*c*_|| is 10 times of the infarct zone in order to approximate diffusion into an infinite space, following Rouillard and Holmes.^17^ Continuity of the chemokine field requires that

(4) $${\left(C_{|X\in \Omega_{b}}\right)}^{+}={\left(C_{|X\in \Omega_{b}}\right)}^{-}.$$

For a spherical and 3D infarct zone of radius *r*_0_, (2) has an analytical solution,

(5) $$C(r)=\left\{\begin{array}{cc} \frac{a_{1}\exp(a_{2}r)-a_{1}\exp(-a_{2}r)}{r}+a_{0}, & r\leq r_{0}\\ \frac{a_{3}\exp(-a_{2}r)}{r}, & r>r_{0}, \end{array}\right.$$

where *a*_0_  =  *k*_c,gen_/*k*_c,deg_, $a_{1}=-\frac{a_{0}(r_{0}+1/a_{2})}{2\exp(a_{2}r_{0})}$, $a_{2}=\sqrt{k_{\text{c,deg}}/D_{c}}$, and $a_{3}=\frac{1}{2}(r_{0}-1/a_{2})\exp(a_{2}r_{0})-a_{1}$.

The fibroblast activation parameters, including cell migration speed, collagen degradation rate, collagen deposition rate, and collagen reorientation rate, are all modulated by the local chemokine concentration *C*(*r*). To compute the activation parameters in the agent‐based model, the local fibroblast activation parameters are assumed to vary linearly with *C* as

(6) $$P_{i}(X)=\left(\frac{P_{i,\max}-P_{i,\min}}{C_{\max}-C_{\min}}\right)\left[C(X)-C_{\min}\right]+P_{i,\min},$$

where *P*_*i*_ is the *i*th rate parameter, *P*_*i*,max_ and *P*_*i*,min_ are the maximum and minimum rates, and *C*_max_, *C*_min_ are the maximum and minimum chemokine concentrations.

#### 2.2.2 Fibroblast migrations regulated by environmental cues

We assume fibroblasts are regulated by local environmental cues.^16^ These include the chemical, mechanical, persistence, and structural cues.^15^, ^16^, ^17^ We further assume the fibroblasts are rigid spheres with radius *R*_cell_, and there is no interaction between fibroblasts. We also use Θ to denote the angles of the fibroblast cells at time t, and *θ* to indicate the collagen fibre angles.

##### Chemical cues

We define the chemokine vector as the product of the chemokine concentration and the outward normal vector at the fibroblast boundary. The chemical cue **v**_*c*_ is obtained from

(7) $$v_{c}=\frac{1}{2\pi }\int_{-\pi }^{\pi }C(r)n_{c}(\Theta )d\Theta ,$$

where **n**_*c*_(Θ) is the unit outward normal vector of the cell surface in the current configuration, *r* is the distance from the boundary of a fibroblast cell to the infarction centre **X**_c_.

##### Mechanical cues

The mechanical cue **v**_*m*_ is defined as

(8) $$v_{m}=\frac{1}{2\pi }\int_{-\pi }^{\pi }\varepsilon_{n}(\Theta )n_{c}(\Theta )\;d\Theta ,$$

where *ε*_*n*_  =  **n**_*c*_·**D** **n**_*c*_ is the normal strain,

$$D=\frac{1}{2}(I-F^{-T}F^{-1})$$

is the Almansi strain tensor, and **I** is the identity matrix.

##### Structural cues

The structural cue **v**_*s*_ is defined as

(9) $$v_{s}=\frac{\int_{-\frac{\pi }{2}}^{\frac{\pi }{2}}N(\theta )f(\theta )\;d\theta }{\int_{-\frac{\pi }{2}}^{\frac{\pi }{2}}N(\theta )d\theta },$$

where *N*(*θ*) is the number of collagen fibres at angle *θ*, **f**(*θ*) is the unit vector in the current configuration defined by **f**  =  **Ff**_0_/||**Ff**_0_||, where $f_{0}=\cos\theta \;c_{0}+\sin\theta \;l_{0}$ is the fibre vector in the reference configuration.

##### Persistence cue

The persistence cue **v**_*p*_ is defined as the fibroblast cell migration velocity

(10) $$v_{p}=S_{\text{cell}}(X)U(\Theta ),$$

where **U**(Θ) is the unit vector along Θ in the current configuration, given by **U**(Θ)  =  **FU**_0_(Θ)/||**FU**_0_(Θ)||, where $U_{0}(\Theta )=\cos\Theta \;c_{0}+\sin\Theta \;l_{0}$.

The fibroblast position at time *t* is tracked from

(11) $$x(t)=\int_{t_{0}}^{t}v_{p}dt.$$

##### Resultant cue

**Figure 2:**
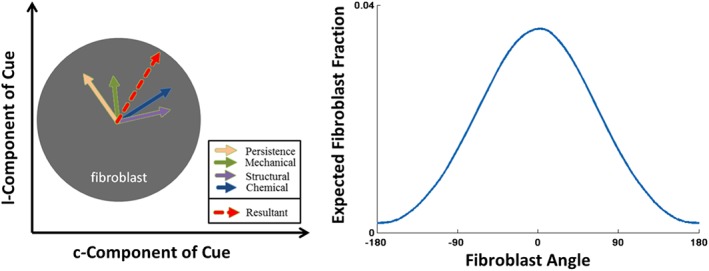
The resultant cue that represents the mean fibroblast direction is computed from a weighted combination of all cues (left). The von Mises distribution of the fibroblast orientation (right)

The fibroblast resultant cue ***ρ*** (Figure 2) is a weighted and normalised sum of all the cues (7) to (10), ie,

(12) $$\rho =\frac{\sum_{i}c_{i}}{\eta +\sum_{i}||c_{i}||},\;c_{i}=\frac{W_{i}}{M_{i}}v_{i},$$

where **v**_*i*_ is the *i*th cue vector, **c**_*i*_ is the *i*th normalised and weighted cue vector, *M*_*i*_ and *W*_*i*_ are *i*th scaling and weighting factors for the *i*th cue as defined in Fomovsky and Holmes,^16^ and *η* is the persistence tuning factor. The orientation of ***ρ*** provides the mean fibre angle $\bar{\Theta }$. We assume that the fibroblasts obey a von Mises distribution

(13) $$\xi_{\text{VM}}(\Theta |\bar{\Theta },\sigma )=\frac{e^{\sigma \cos(\Theta -\bar{\Theta })}}{I_{0}(\sigma )},\;\sigma^{2}=-2\ln||\rho ||,$$

where *I*_0_ is the Bessel function of the first kind of order zero

$$I_{0}(\sigma_{0})=\frac{1}{\pi }\int_{0}^{\pi }\exp(\sigma_{0}\cos\Theta )d\Theta .$$

#### 2.2.3 Remodelling of the collagen fibre structure

The local collagen fibre distribution in the current configuration is remodelled by fibroblasts in the following way.

1. Deposition and degradation: The total collagen fibre number at time t is
  (14) $$N_{\text{tot}}=\int_{-\pi /2}^{\pi /2}N(\theta )d\theta .$$
  The rate of change of the number N(θ) of collagen fibres in the θ direction is determined from
  (15) $$\frac{\partial N(\theta )}{\partial t}=k_{\text{cf,gen}}(C)\beta \pi R_{\text{cell}}^{2}\delta (\Theta -\theta )-k_{\text{cf,deg}}(C)N(\theta ),$$
  where β is the maximum collagen fibre number per unit area, R_cell_ is the radius of the fibroblast cell, δ is the delta function to ensure that new collagen fibres are aligned with the fibroblast migration direction, and k_cf,gen_ and k_cf,deg_ are the rates of generation and degradation of collagen fibres, respectively.
2. Rotation: The angle of a collagen fibre changes according to
  (16) $$\frac{\partial \theta }{\partial t}=\left\{\begin{array}{c} k_{\text{cf,rot}}(C)||\sin(\Theta -\theta )||,\;\Theta -\theta \in (-\frac{\pi }{2},0)\cup (\frac{\pi }{2},\pi )\\ -k_{\text{cf,rot}}(C)||\sin(\Theta -\theta )||,\;\Theta -\theta \in (0,\frac{\pi }{2})\cup (\pi ,\frac{3\pi }{2}), \end{array}\right.$$
  where k_cf,rot_ is the rate of rotation estimated from (6).

We assume that the material properties are the same for **f** and −**f**, so we only need to consider values of θ  ∈  ( − π/2,π/2). To achieve this, the following transformation is used:

(17) $$\left\{\begin{array}{c} \theta ⇐\theta ,\;\theta \in (-\frac{\pi }{2},\frac{\pi }{2})\\ \theta ⇐\theta -\pi ,\;\theta >\frac{\pi }{2}\\ \theta ⇐\theta +\pi ,\;\theta <-\frac{\pi }{2}. \end{array}\right.$$

The deformed and remodelled fibre structure needs to be pushed back to the reference configuration,^22^, ^23^ ie,

(18) $$f_{0}(\theta )=\frac{F^{-1}f(\theta )}{\left||F^{-1}f(\theta )|\right|}.$$

#### 2.2.4 Upscaling the fibre structure to the tissue level

The updated volume fraction Φ_cf,t_ is

(19) $$\Phi_{\text{cf},t}=\frac{N_{\text{tot}}}{N_{\text{tot}}^{0}}\Phi_{\text{cf}}^{0},$$

where $\Phi_{\text{cf}}^{0}$ and $N_{\text{tot}}^{0}$ are the volume fraction and numbers of the fibres in the reference configuration.

The fibre orientation density function, when pushed back to the reference configuration, is estimated from

(20) $$\phi_{t}(\theta )=\pi \frac{N(\theta )}{N_{\text{tot}}}.$$

In the initial reference configuration, we assume that the fibres have π‐periodic von Mises distribution^24^, ^25^:

$$\phi_{0}(\theta )=\frac{e^{\sigma_{0}\cos(\theta_{0}-{\bar{\theta }}_{0})}}{I_{0}(\sigma_{0})},$$

where σ_0_ is the initial concentration parameter, fitted from experimental observation,^17^ and ${\bar{\theta }}_{0}$ is the initial mean angle of the collagen fibre structure.

### 2.3 Constitutive laws for myocardium

#### 2.3.1 Modified HO model with fibre orientation density function

For the remote healthy myocardium (r(**X**)  >  r_0_), we use the standard HO model,^14^ ie,

(21) $$\Psi =\Psi_{m}+\Psi_{\text{cf}},$$

where

(22) $$\Psi_{m}=\frac{a}{2b}\left\{\exp\;\left[b(I_{1}-3\right]-1\right\},\;\Psi_{\text{cf}}=\frac{a_{\text{cf}}}{2b_{\text{cf}}}\left\{\exp\left[b_{\text{cf}}{\left(I_{4}(\theta )-1\right)}^{2}\right]-1\right\},$$

in which a,b,a_cf_, b_cf_ are material parameters, and I_1_, I_4_ are the invariants of the right Cauchy–Green tensor **C**  =  **F** ^T^ **F**,

(23) $$I_{1}=C:I,\;I_{4}={\vec{f}}_{0}(\theta )\cdot C{\vec{f}}_{0}(\theta ).$$

Here, “:” denotes a double contraction.

To describe the mechanical behaviour of the infarcted tissue (r(**X**)  ≤  r_0_), we modify the HO model as

(24) $$\Psi =\Phi_{m,t}\Psi_{m}+\Phi_{\text{cf},t}\Psi_{\text{cf}},$$

where Φ_m,t_(  =  1 − Φ_cf,y_) is the volume fraction of the ground matrix, Ψ_m_ is the same as in (22) but Ψ_cf_ is changed to

(25) $$\Psi_{\text{cf}}=\frac{1}{\pi }\frac{a_{\text{cf}}}{2b_{\text{cf}}}\int_{\Sigma }\left\{\exp\left[b_{\text{cf}}{\left(I_{4}(\theta )-1\right)}^{2}\right]-1\right\}\phi_{t}(\theta )\;d\theta .$$

Notice that the integration in (25) is over a domain Σ in which the fibres are in tension, not ( − π/2,π/2). This is because collagen fibres bear load only when they are stretched. We will address this point in Section 2.3.2.

A rule‐based myocardium fibre generation algorithm^21^, ^26^) is adopted to describe the local mean fibre angle. We first calculate the normalised distance parameter d for an arbitrary material point **X** as

(26) $$d=\frac{d_{\text{endo}}}{d_{\text{endo}}+d_{\text{epi}}},$$

where d_endo_, d_epi_ are the distances from **X** to the endocardial and epicardial surfaces. Then, the local mean fibre angle ${\bar{\theta }}_{0}$ at **X** is defined as

$${\bar{\theta }}_{0}=\theta_{\max}(1-2d),$$

where $\theta_{\max}=\pi /3$, because it has been observed that the mean angle of fibres in a healthy left ventricle rotates transmurally across the heart wall from π/3 at the endocardial surface to −π/3 at the epicardial surface.^27^

#### 2.3.2 Exclusion of the compressed fibres

**Figure 3:**
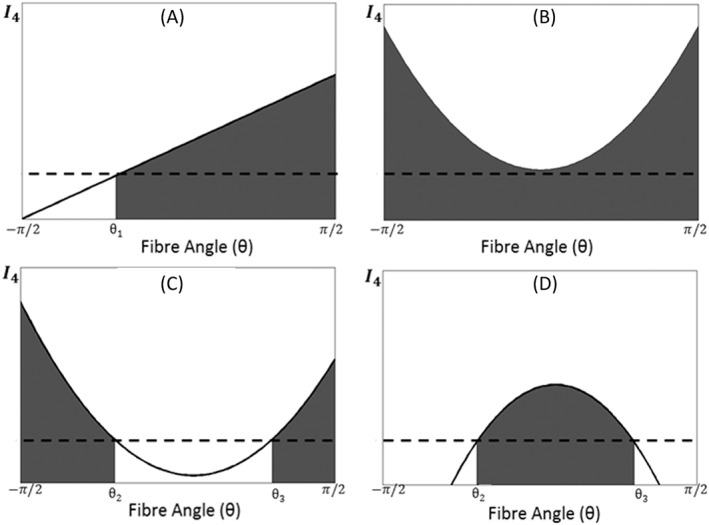
The shaded areas show the range of Σ of stretched collagen fibres within $-\frac{\pi }{2}<\theta <\frac{\pi }{2}$ for selected scenarios (dash line denotes I_4_  =  1): A, Case 1, C_23_  >  0. B, Case 2, C_33_ − 1  >  0,Δ  ≤  0. C, Case 2, C_33_ − 1  >  0,Δ  >  0. D, Case 2, C_33_ − 1 < 0,Δ  >  0

To exclude the compressed fibres, we choose Σ in (25) so that

(27) $$I_{4}-1\equiv (C_{22}-1)\cos^{2}\theta +2C_{23}\sin\theta \cos\theta +(C_{33}-1)\sin^{2}\theta >0,$$

where C_ij_ are the Cartesian components of **C**. The inequality (27) gives the range of Σ for the stretched collagen fibres in the following two cases, as exemplified in Figure 3.

- Case 1: C_33_  =  1
  (28) $$\Sigma =\left\{\begin{array}{cc} \left(\theta_{1},\frac{\pi }{2}\right) & C_{23}>0\\ \left(-\frac{\pi }{2},\theta_{1}\right) & C_{23}<0\\ \left(-\frac{\pi }{2},\frac{\pi }{2}\right) & C_{23}=0,\;C_{22}>1\\ \emptyset & C_{23}=0,\;C_{22}<1, \end{array}\right.$$
  where $\theta_{1}=\arctan\left[\left(1-C_{22})/(2C_{23}\right)\right]$.
- Case 2:
  (29) $$\Sigma =\left\{\begin{array}{cc} \left(-\frac{\pi }{2},\frac{\pi }{2}\right) & C_{33}-1>0,\;\Delta \leq 0\\ \left(-\frac{\pi }{2},\theta_{2}\right)\cup \left(\theta_{3},\frac{\pi }{2}\right) & C_{33}-1>0,\;\Delta >0\\ \left(\theta_{2},\theta_{3}\right) & C_{33}-1<0,\;\Delta >0\\ \emptyset & C_{33}-1<0,\;\Delta <0, \end{array}\right.$$
  where Δ  =  (2C_23_)^2^ − 4(C_33_ − 1)(C_22_ − 1), and θ_2_ and θ_3_ are the roots of I_4_  =  1 in (27).
  (30) $$\sigma =-pI+\Phi_{m,t}\;a\;\exp[b(I_{1}-3)]B+\frac{2a_{\text{cf}}}{\pi }\Phi_{\text{cf},t}\int_{\Sigma }(I_{4}-1)\exp[b_{\text{cf}}{\left(I_{4}-1\right)}^{2}]\phi (\theta )u(\theta )⊗u(\theta )d\theta ,$$
  where **B**  =  **F** **F** ^T^ is the left Cauchy‐Green deformation tensor. All the constitutive parameters are given in Table 2.

**Table 2:** Fitted material parameters for myocardium from Rouillard and Holmes^17^ and Omens et al^28^

Initial volumetric fractions	$\Phi_{m,t_{0}}=0.97$	$\Phi_{\text{cf},t_{0}}=0.03$
Initial concentration parameter	*σ*_0_ = 9.10
Matrix parameters	*a* = 2.28 kPa	*b* = 1.8
Collagen parameters	*a*_cf_ = 132.0 kPa	*b*_cf_ = 3.45

#### 2.3.3 Change of basis from Cartesian to local coordinates

It is convenient to transform the description of fibre orientation between the global Cartesian coordinates and the local polar basis vectors, so that right Cauchy–Green tensor are given as

(31) $$C^{*}=Q\cdot (F^{T}F)Q^{T},$$

where **Q** is the rotation tensor from Cartesian basic vectors to the local polar basis vectors in the reference configuration.

### 2.4 The coupled agent‐based and FE model

The LV diastolic dynamics is simulated using a coupled agent‐based FE LV model. We assume that mechanical cues are based on the end‐diastolic state and the remodelling only occurs in the MI region. The FE model is solved by using the FE software FEAP,^29^ in which the incompressibility is enforced with a penalty method.^30^ Specifically, we consider the myocardium as a quasi‐incompressible material. Using the multiplicative decomposition of the deformation gradient **F**,

$$F=J^{1/3}\bar{F};\;C=J^{2/3}\bar{C},$$

where *J*  =  det(F), we write the strain energy function Ψ as

(32) $$\Psi =\Psi_{v}(J)+{\bar{\Psi }}_{m}(\bar{C})+{\bar{\Psi }}_{\text{cf}}({Ī}_{4}),$$

where $\bar{C}={\bar{F}}^{T}\bar{F}$, and ${\bar{\Psi }}_{m}$, ${\bar{\Psi }}_{\text{cf}}$ are isochoric contributions of the matrix and fibres, respectively, expressed in terms of the modified invariants, ${Ī}_{1}(=\bar{C}:I)$ and ${Ī}_{4}(=f_{0}\cdot \bar{C}f_{0})$,

(33) $$\begin{array}{ccc} {\bar{\Psi }}_{m} & =\Phi_{m,t}\frac{a}{2b}\left[\exp\;b({Ī}_{1}-3)-1\right], & \\ {\bar{\Psi }}_{\text{cf},t} & =\Phi_{\text{cf},t}\frac{1}{\pi }\frac{a_{\text{cf}}}{2b_{\text{cf}}}\int \left[\exp\;b_{\text{cf}}{\left({Ī}_{4}(\theta )-1\right)}^{2}-1\right]\phi_{t}(\theta )\;d\theta . \end{array}$$

The volumetric penalty term is chosen to be

$$\Psi_{v}=\frac{k}{2}{\left(J-1\right)}^{2}.$$

The Cauchy stress is then

(34) $$\begin{array}{ccc} \sigma & =k(J-1)I+\Phi_{m,t}\;a\;\exp[b({Ī}_{1}-3)]\text{dev}\bar{b} & \\ & \;+\frac{1}{\pi }\Phi_{\text{cf},t}\int_{\Sigma }2a_{f}({Ī}_{4}-1)\exp[b_{f}{\left({Ī}_{4}-1\right)}^{2}]\phi_{t}(\theta )\;\text{dev}(f⊗f)d\theta , \end{array}$$

where $\bar{b}=\bar{F}{\bar{F}}^{T},$ and the operator dev(•) is defined as

$$\text{dev}(•)=(•)-\frac{1}{3}[(•):I]I.$$

**Figure 4:**
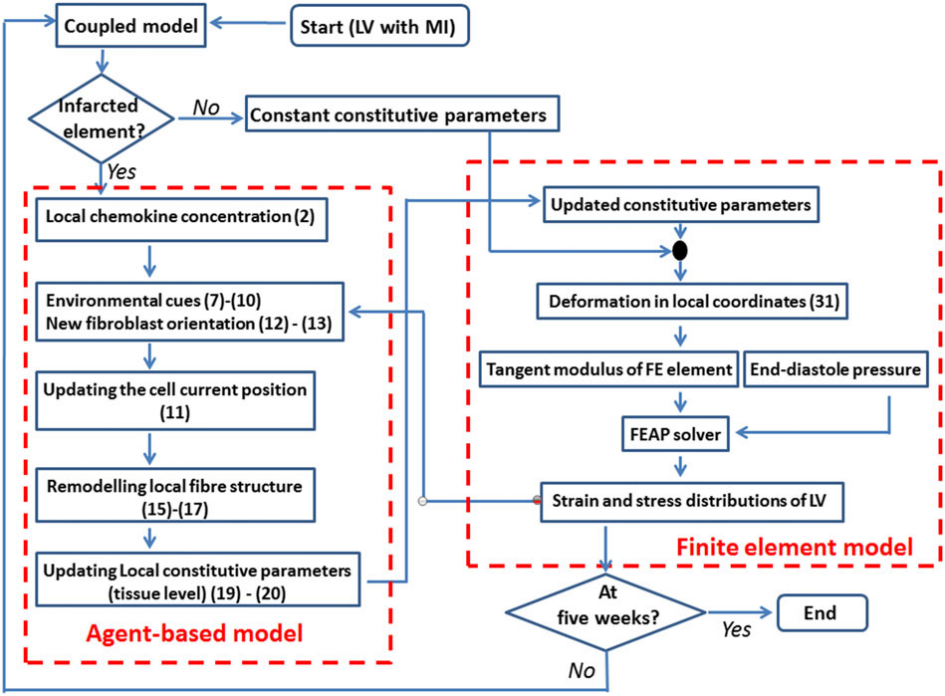
Flowchart for the coupled agent‐based and FE LV model computation. FE, finite element; LV, left ventricle; MI, myocardial infarction

The Hu‐Washizu mixed variational approach is used to solve the FE equations to avoid locking,^29^ as briefly listed in Appendix A. The computational flowchart is shown in Figure 4.

## 3 RESULTS

### 3.1 MI healing case studies

**Figure 5:**
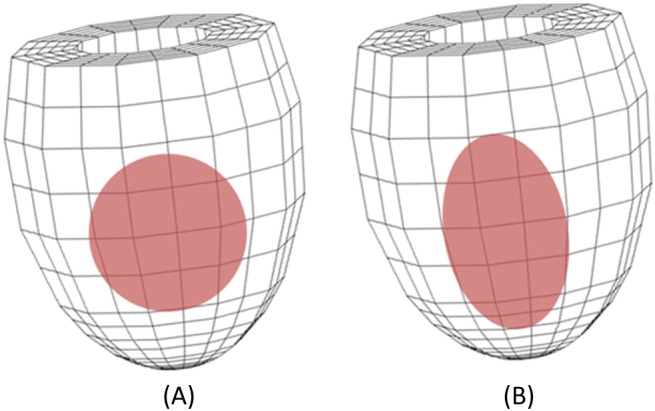
The finite element models of A, a transmural circular cryoinfarct with r_0_≈5.5 mm and **X**_**c**_  =  (10.12,2.82,0.81), and B, a transmural elliptical cryoinfarct (with long axis ≈ 15 mm, short axis ≈ 5 mm, and **X**_**c**_  =  (10.12,2.82,0.81), in the anterior wall

To validate the model, we follow the experimental study of the infarcted LV of rats by Fomovsky et al,^31^ in which cryoinfarctions were created by sewing steel cylinders filled with liquid nitrogen into tissues under the epicardial surfaces of rat ventricles. After weeks of healing, the hearts were harvested to study the collagen structures at the surfaces of the infarcted zones. We select two cases from Fomovsky et al^31^ to simulate. In the first case, a transmural circular MI is induced as shown in Figure 5A, and in the second case, a transmural elliptical MI is as shown in Figure 5B, each in the mid‐ventricular wall.

We modelled the healing process for 5 weeks while the mature scar was formed and the ventricular filling pressure increased.^31^ A linearly increasing end‐diastolic pressure profile is assumed, with values of 12 mmHg at time 0 to 18 mmHg at 5 weeks. The LV base was constrained in the longitudinal direction, but free movements in the radial and circumferential directions were allowed. The model parameters are listed in Table 2, taken from Fomovsky et al.^31^

### 3.2 Evolution of the fibre structure post‐MI

**Figure 6:**
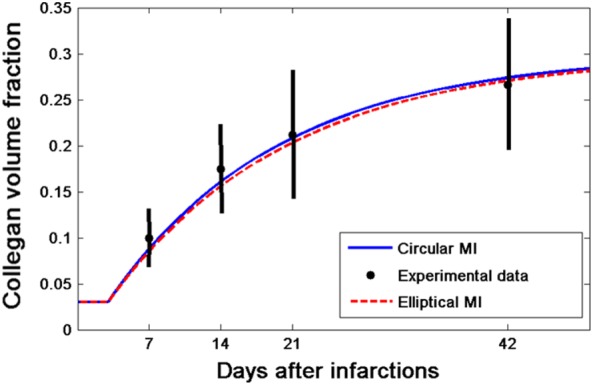
Comparison of estimated infarct collagen volume evolution with the measurements^16^ (black dots with error bars). MI, myocardial infarction

The simulated collagen accumulations for both the circular and elliptical MI shapes in the infarct centre at 0, 1, 2, and 5 weeks post‐MI are shown in Figure 6. The volume fraction increases from 3% to around 28%. This agrees well with the measured data.^16^ We note that the difference of the volume fraction increase is very little between the different MI shapes (28.2% for circular MI vs 28.0% for elliptical MI after 5 weeks).

**Figure 7:**
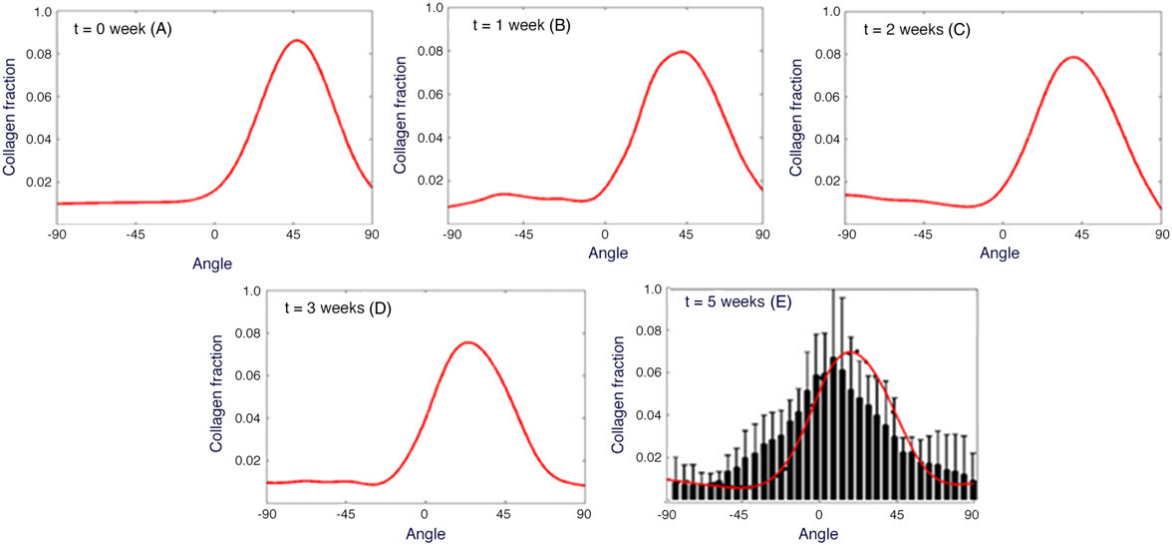
The evolution of collagen fibre structure for a circular myocardial infarction (MI): A,‐D, 0, 1, 2 and 3 weeks. E, The overlap of estimated fibre structure with the experimental measurements 5 weeks post‐MI^16^

The distributions of the collagen fibres at different weeks post‐MI for the circular case are illustrated in Figure 7. At 5 weeks post‐MI, the simulated result is also compared with the corresponding measurement.^16^ Given the large error bars in the measurements, there is a reasonable agreement in terms of the overall distribution and the maximum collagen fibre fraction predicted.

**Figure 8:**
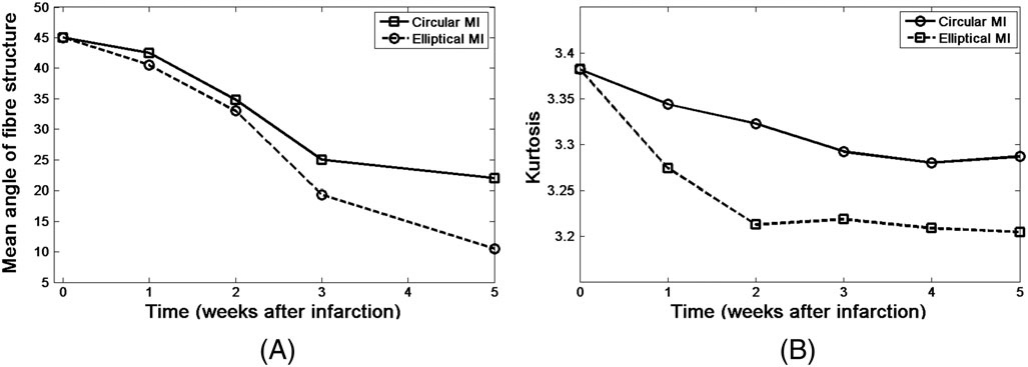
A, The mean angle for the circular and elliptical myocardial infarctions (MIs). B, The kurtosis of the fibre structure

Figure 8A shows that for both MI shapes, the mean angle of collagen fibre families at 20% of the distance from the epicardium to the endocardium surface decreases significantly at the fifth week (from the initial 45° to 22.5° for the circular MI, and to 10° for the elliptical MI).

**Figure 9:**
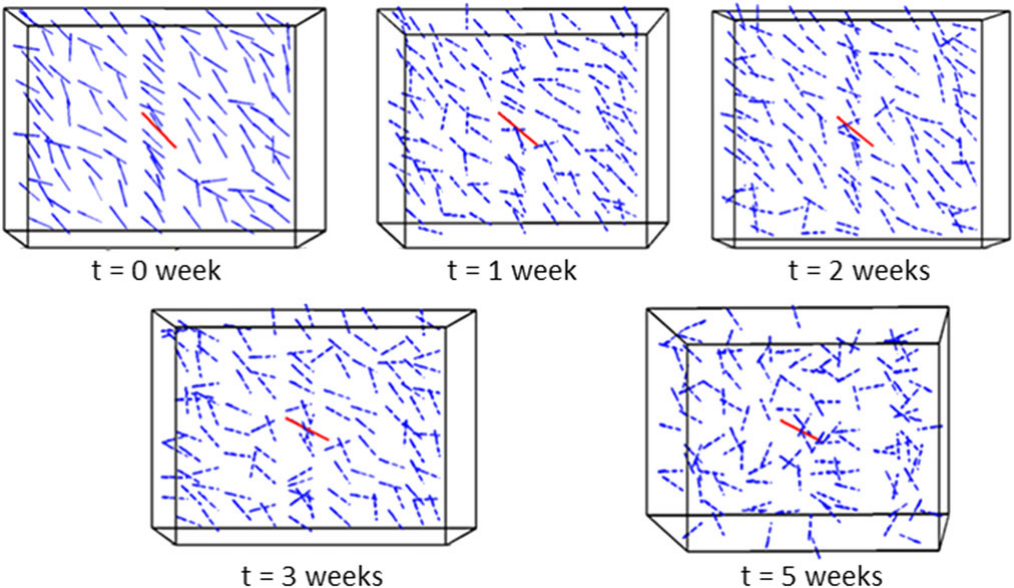
Evolution of the collagen structure at the epicardial surface of the circular infarcted zone. The red lines define the mean angles of the overall fibre structure, while the blue lines indicate the mean fibre vectors over smaller local clusters

As the anisotropy in collagen fibre structure is a critical determinant for the pump function of LV,^17^, ^31^ we use the kurtosis of the collagen fibre structure to describe the anisotropy level, which is defined as

(35) $$\text{Kurt}(\theta )=\frac{E\left[{\left(\theta -\bar{\theta }\right)}^{4}\right]}{E{\left[{\left(\theta -\bar{\theta }\right)}^{2}\right]}^{2}},$$

where E(•) is the expectation of •, and $\bar{\theta }$ is the mean fibre angle. A smaller value of kurtosis indicates a lower level of anisotropy. Figure 8B shows that the anisotropy level at the infarcted region decreases initially but eventually reaches a plateau. It is interesting to see that although the fibre volume fraction is similar for both the circular and elliptical MI shapes, the distribution of the fibres is quite different. The evolution of the fibre structure for the elliptical MI is faster than that for the circular MI. The anisotropy level of the fibre structure, in terms of the kurtosis, decreases from the initial value of 3.38 to 3.29 for the circular MI, and to 3.21 for the elliptical MI. In general, the elliptical MI causes greater changes in terms of the mean fibre angle and kurtosis over the healing time. However, in both cases, the volume fraction increased to a stable level of around 28% (Figure 6) at the fifth week. The decreased anisotropy of the fibre distribution over time is better shown in Figure 9, where the local fibre structure near the epicardial surface at different weeks post‐MI is illustrated. Note that to make the fibre structure visible, we have used the averaged fibre vectors of small local clusters, each consisting of hundreds of the simulated fibre vectors.

### 3.3 Evolution of the stress and strain post‐MI

**Figure 10:**
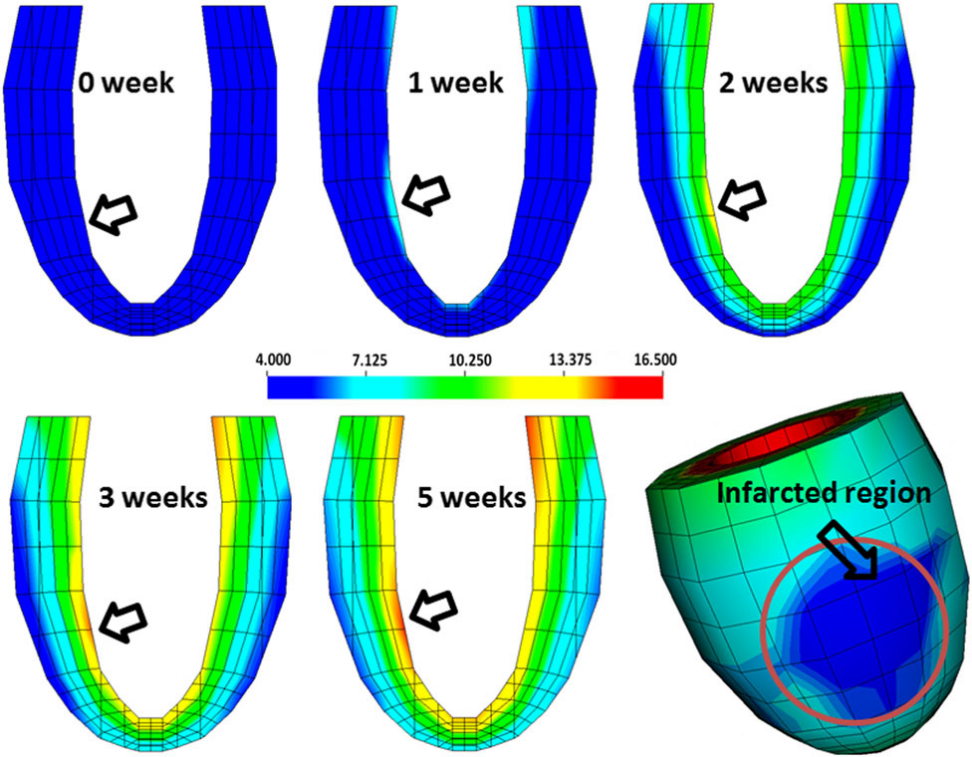
Evolution of the first principal stress distribution for the circular myocardial infarction (MI); its location is indicated by the arrow. The bottom right‐hand figure is a three‐dimensional (3D) plot at 5 weeks post‐MI

The first principal stress distribution for the circular MI at different times post‐MI is illustrated in Figure 10, where the infarcted region is indicated by an arrow. It is evident that the stress distribution is greatly influenced by the evolution of the local collagen fibre structure. The stress increases at the endocardial surface and gradually smears out towards the epicardial surface. The local stress level around the MI region is caused by collagen accumulation and reorientation, but the overall stress also increases due to the increased end‐diastolic pressure.

**Figure 11:**
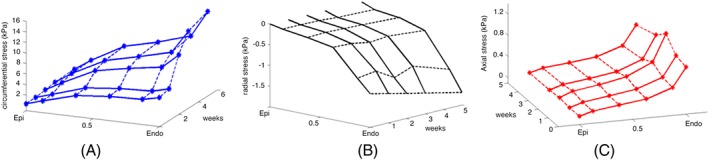
Evolution of A, radial stress; B, circumferential stress; C, longitudinal stress, for the circular myocardial infarction (MI)

The evolution of the stress level with time is shown in Figure 11, and the corresponding mean stress values at the infarct centre are also listed in Table 3. During the healing process, the circumferential stiffness is reinforced by the newly deposited collagen fibres. Hence, the maximum circumferential stress increases dramatically from 4.3 to 15.1 kPa. The local longitudinal stress has not changed much (from 0.78 to 0.76 kPa at 5 weeks). This is because most of the collagen fibre remodelling happens in the circumferential direction. The elevated circumferential stress level around the MI region can lead to further adverse remodelling in the heart, such as myocyte apoptosis, because the local abnormal stress might cause cell degradation.^32^

**Table 3:** Parameter values related to fibroblast dynamics and collagen remodelling

Fibroblast Dynamics				
Radius of fibroblast cell	*R*_cell_	5	μm	
Maximum collagen fibre number per volume	*β*	14000	μm^−3^	
Chemokine diffusion coefficient	*D*_*c*_	1.6	μm^−3^s^−1^	
Chemokine degradation rate	*k*_c,deg_	0.001	s^−1^	
Chemokine generation rate	*k*_c,gen_	0.01	nms^−1^	
Persistence tuning factor	*η*	0.175		
Persistence cue weight factor	*W*_p_	0.333		
Structural cue weight factor	*W*_s_	0.167		
Mechanical cue weight factor	*W*_m_	0.167		
Chemical cue weight factor	*W*_c_	0.167		
**Collagen Remodelling**				
		Quiescent (Min)	Activated (Max)	
Collagen degradation rate coefficient	*k*_cf,deg_	2.4E‐4	2.5E‐3	h^−1^
Collagen generation rate coefficient	*k*_cf,gen_	7.5E‐4	7.3E‐2	*%*volume· h^−1^
Chemokine fibre rotation generation rate	*k*_cf,rot_	5	5	deg· h^−1^

The stress distribution for the elliptical MI is similar to that of the circular MI, but the absolute stress levels are slightly different, as shown in Table 3.

**Figure 12:**
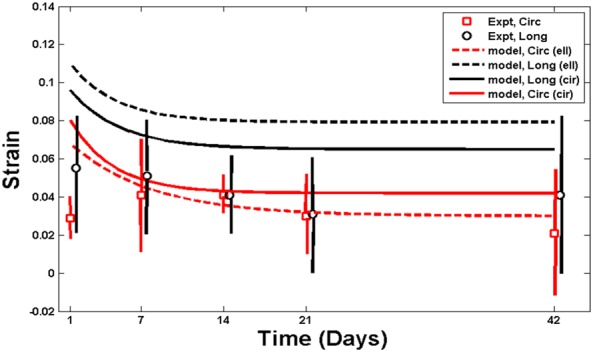
Strain evolution over time: preferential accumulation in the circumferential direction reduces the anisotropy. The predicted strain trends agree reasonably with the experimentally measured strains 5 weeks after cryoinfarction^16^

Fomovsky et al^31^ also measured the evolution of strain during the healing process in the epicardial surface of the infarcted region. They found that the circumferential strain in the infarct region continuously decreases with an average decrease of 65%, and an average decrease of 13% for the longitudinal strain at 6 weeks. Our simulation results give similar trends, as shown in Table 4 and in Figure 12 for both MI shapes. For example, in the case of the circular MI, the strain shows an average decrease of 49% in the circumferential strain (0.03 ± 0.01 at 0 week, 0.04 ± 0.024 at 1 week, 0.042 ± 0.01 at 3 weeks, 0.03 ± 0.02 at 4 weeks, and 0.022 ± 0.02 at 5 weeks), and an average decrease of 35% in the longitudinal strain at 5 weeks.

**Table 4:** Comparison of the mean stresses at the infarct centre post‐MI

	Circumferential Stress, kPa		Longitudinal Stress, kPa	
	Circular MI	Elliptical MI	Circular MI	Elliptical MI
0 week	4.3	4.5	0.78	0.78
1 week	5	5.8	0.82	0.80
2 weeks	10.2	8.8	1.17	0.84
3 weeks	13.7	13.7	0.91	0.61
5 weeks	15.1	16.3	0.76	0.49

Abbreviation: MI, myocardial infarction.

The evolution of the strains at the endocardial surface of the elliptical MI is similar to that of the circular MI (Figure 12). Both circumferential and longitudinal strains decrease to the homeostatic state after the healing process, with 31% decrease in the circumferential strain and 43% decrease in the longitudinal strain.

### 3.4 Influence of different cues

**Figure 13:**
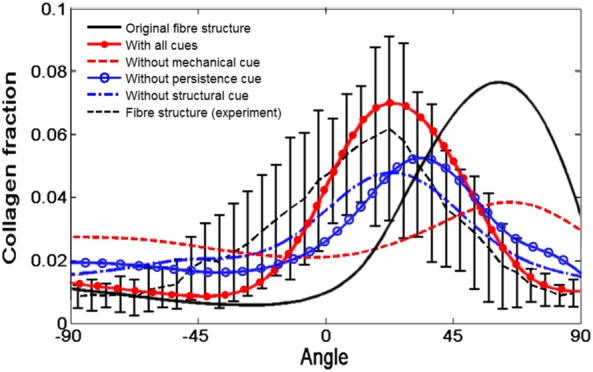
Fibre structure at 5 weeks post‐MI, with all the environmental cues (red solid with filled circle), without the mechanical cue (red dashed), without the structural cue (blue dash dot), without the persistence cue (blue solid with hollow circle). The measured data in Fomovsky and Holmes^16^ are shown as a black dashed curve with error bars. The distribution at week 0, estimated from a healthy subject^14^, ^17^ is also shown for comparison (black solid). MI, myocardial infarction

We now isolate the effect of different cues by switching off individual cues in turn. The distributions of the collagen fibres are shown in Figure 13. The mechanical cue was suggested to be the most important factor in regulating the collagen alignment.^17^ This is confirmed in our study. When the mechanical cue is switched off, the collagen fraction in the infarcted region decreases more quickly, and the mean angle of the fibre structure at the endocardial surface remains 60°, which is similar to that in healthy heart tissue.^14^ In other words, with the mechanical cue, the mean angle of the fibre structure at the endocardial surface reduces from 60° to 25°, which is closer to the experimentally measured fibre value.^16^ The effects of other cues are less prominent. For instance, the mean angles of the fibre structure at the endocardial surface change from 37° to 25° if the persistence cue is turned off; from 25° to 21° if the structure cue is turned off. The fact that the mechanical cue plays the most important role in the post‐MI fibre distribution is also confirmed in a recent experimental study.^33^

## 4 DISCUSSION

Our model is the first to couple an agent‐based model and a 3D idealised LV model with local MI and is a significant extension of the previous study based on a 2D FE slab.^34^ In particular, a modified HO constitutive law is employed to simulate the mechanical behaviour of the myocardium post‐MI, where fibre dispersion due to wound healing needs to be accounted for. To exclude the effects of the compressed fibres, an analytical fibre switch is implemented in this FE LV model.

To use a model that includes the evolving collagen fibre structure post‐MI requires one to estimate many material parameters at different length scales. In this study, the model parameters are taken from published cryoinfarction measurements,^16^ and the predictions of our model showed good quantitative agreement. The only parameter not based on the measurements is the persistence tuning factor *η* (Table 2). To see how much effect this has, we also ran the model with the value of *η* increased. Our results (not shown) suggest that an increased *η* leads to a greater standard deviation *σ*, reducing the randomness of the new fibroblast angles through (13). The fibre structure reached the steady state faster, and matured at around 4 weeks when *η* was doubled, which is one week earlier than by using the default value of *η*. The final fibre structures, however, are very similar.

Environmental cues, such as the chemical, mechanical and persistence cues, play important roles in regulating collagen fibre structures during MI healing. Our results show the poorest agreement with measurements if the mechanical cue is switched off. According to Zimmerman's experimental observation on the effect of initial collagen fibre structure,^35^ the orientations of existing collagen fibres act as the template for alignments of new fibres during infarction healing process. This agrees with our model prediction when the structural cue is switched off. In this case, the collagen structure is more isotropic after scar formation; however, the remodelled mean angle of the fibre structure does not change much, with or without the structural cue. If the persistence cue is switched off, the distribution of the fibre structure is similar, but the shift of the mean angle is smaller, and the evolution is much slower than that in the experimental measurement.^16^ McDougall et al^36^ suggested that the distribution of the chemical gradient established by the chemokines generated around dermal wounds, determines patterns of local collagen alignment. In our simulations, different chemokine distributions are realised through the MI shapes; the chemokine field is mostly flat inside the infarction, but with a sudden drop near the infarct border. The influences of chemical gradient are greater in the region near the border. Therefore, the mean angle and kurtosis of the fibre distributions for different infarct shapes are different. Our simulations are also supported by the observations in Rouillard and Holmes.^17^

Finally, we mention the limitations of this study. Firstly, the transmural fibroblast migration is not considered. Secondly, our model geometry is based on an idealised half ellipsoid and without active contraction. Therefore, the mechanical cue is simplified. Thirdly, we have not included volumetric growth in our model, and therefore, effects such as wall thinning are not included here. Furthermore, no transient chemokine concentration is included. Finally, we understand that MI in the human heart may involve different biological processes and parameters compared with cryoinfarction in the rat heart. Estimation of these parameters for patient‐specific human hearts post‐MI, therefore, remains a daunting task. These limitations may affect the quantitative findings presented in the paper because physiologically correct anatomy and microstructure affect the stress state in the heart and therefore the remodelling phase. However, we believe our agent‐based approach for the time‐dependent density growth and fibre distribution is a step forward towards cellular‐based modelling of MI.

## 5 CONCLUSION

We have developed an agent‐based MI model that takes into consideration collagen remodelling with a 3D LV model in diastole. In this model, the evolution of the microstructure of collagen fibres is simulated under the framework of an agent‐based model based on the statistical behaviour of cellular movements. Then, the microstructure change is upscaled into the tissue level to describe the material properties of infarcted heart tissues. To describe the remodelled material properties of myocardium in the infarcted region, a collagen fibre tension‐compression “switch” is incorporated in the FE LV model for the first time. The time‐dependent model also captures the interaction and information exchange processes between the mechanical behaviour and collagen tissue remodelling guided by various external cues. The model results show similar trends in collagen accumulation and collagen alignment to those found in experimental measurements of infarct healing in the rat heart over 5 weeks. The shapes and the locations of the infarctions could affect the local collagen accumulation and the LV dynamics. For example, the mean angle of the fibre structure decreases from about 45^*o*^ to 22.5^*o*^ near the endocardial surface for the circular infarction but decreases less in the elliptical infarction. Stresses are affected in both the circular and the elliptical infarctions. The reduction of the circumferential strains at 5 weeks post‐MI agrees well with the experimental observations. With further development, we expect that this agent‐based homogenized approach could provide useful insights for the clinical practice of MI patient management.

## APPENDIX A FINITE ELEMENT IMPLEMENTATION USING FEAP

We use the mixed variational approach in FEAP to solve the mechanical behaviour of nearly incompressible soft tissue that is free from locking.^29^ For each element, FEAP uses the Hu‐Washizu variational principle

(A1) $$\Pi (u,\hat{\sigma },\hat{\epsilon })=\frac{1}{2}\int_{\Omega }{\hat{\epsilon }}^{T}D\hat{\epsilon }\;d\Omega +\int_{\Omega }{\hat{\sigma }}^{T}(\nabla^{\left(s\right)}u-\hat{\epsilon })d\Omega -\int_{\partial \Omega^{u}}T^{T}(u-u^{b})d\Omega -\int_{\partial \Omega^{\sigma }}u^{T}t^{b}d\Omega ,$$

where $\hat{\sigma }$, $\hat{\epsilon }$ are the element ordered vectors of stress and strain tensors, **u** is the displacement vector, **D** is the ordered matrix of tangent moduli tensor D, **t** is the traction, ∇^(*s*)^ **u** is the symmetric part of ∇**u**, **t** ^*b*^ is the traction on the force boundary *∂*Ω^***σ***^, **u** ^*b*^ is the displacement vector on the displacement boundary *∂*Ω^**u**^ (see Taylor^29^ for details about the definition of an ordered vector). Splitting the variables into the deviatoric and volumetric parts, we can write (A1) as follows:

(A2) $$\begin{array}{ccc} \Pi (u,\hat{\sigma },\hat{\epsilon }) & =\frac{1}{2}\int_{\Omega }{\hat{\epsilon }}^{T}D_{\text{dev}}\hat{\epsilon }\;d\Omega +\int_{\Omega }{\hat{\sigma }}_{\text{dev}}^{T}(\nabla^{\left(s\right)}u_{\;\text{dev}}-{\hat{\epsilon }}_{\text{dev}})d\Omega & \\ & \;+\frac{1}{2}\int_{\Omega }{\hat{\epsilon }}^{T}D_{\text{vol}}\hat{\epsilon }\;d\Omega +\int_{\Omega }{\hat{\sigma }}_{\text{vol}}^{T}(\nabla^{\left(s\right)}u_{\;\text{vol}}-{\hat{\epsilon }}_{\text{vol}})d\Omega \\ & \;-\int_{\partial \Omega^{u}}t^{T}(u-u^{b})d\Omega -\int_{\partial \Omega^{\sigma }}u^{T}t^{b}d\Omega , \end{array}$$

where

$${\left(•\right)}_{\text{dev}}=I_{\text{dev}}(•),\;{\left(•\right)}_{\text{vol}}=I_{\text{vol}}(•),$$

and

$$I_{\text{dev}}=I-\frac{1}{3}{\text{mm}}^{T},\;I_{\text{vol}}=\frac{1}{3}{\text{mm}}^{T},$$

in which I is a 6 × 6 identity matrix, $m^{T}=\left[1\;1\;1\;0\;0\;0\right]$, representing a rank two identity tensor.

The variables are discretised using

(A3) $$\begin{array}{ccc} u(\xi ) & =N(\xi )u(t), & \\ \sigma (\xi ) & =\varphi_{\alpha }(\xi )\sigma (t),\\ \epsilon (\xi ) & =\psi_{\alpha }(\xi )\epsilon (t), \end{array}$$

where ***ξ*** is the natural coordinates, **u**(*t*),***σ***(*t*),***ϵ***(*t*) are the nodal displacement, stress, and strain variables, and *N*(***ξ***), *φ*_*α*_(***ξ***),*ψ*_*α*_(***ξ***) are shape functions, chosen to be the same eight‐node shape function in our simulations.

It remains to derive the fourth‐order tangent stiffness matrix D, which is

(A4) $$D=J^{-1}FFFF𝒞,$$

where 𝒞 is derived from the derivative of the second Piola‐Krirchoff stress **P**,

(A5) $$𝒞=2\frac{\partial P}{\partial C}=2\frac{\partial }{\partial C}\left[2\frac{\partial \Psi }{\partial C}\right]=4\left(\frac{\partial^{2}\Psi_{v}}{\partial C^{2}}+\frac{\partial^{2}{\bar{\Psi }}_{m}}{\partial C^{2}}+\frac{\partial^{2}{\bar{\Psi }}_{\text{cf}}}{\partial C^{2}}\right).$$

For the given strain energy function (33), we have

(A6) $$\begin{array}{ccc} D & =k\left\{\left(2J-1)I-(J-1)(I_{\left(ikjl\right)}+I_{\left(iljk\right)}\right)\right\} & \\ & \;+\Phi_{m,t}\frac{2a\gamma }{J}(b\left[-\frac{1}{3}{Ī}_{1}I+\bar{b}\right]⊗\left[-\frac{1}{3}{Ī}_{1}I+\bar{b}\right]\\ & \;-\frac{1}{3}I⊗\left(-\frac{1}{3}{Ī}_{1}I+\bar{b}\right)+\frac{1}{6}{Ī}_{1}(I_{\left(ikjl\right)}+I_{\left(iljk\right)})\\ & \;-\frac{1}{3}(\bar{b}⊗I))\\ & \;+\Phi_{\text{cf},t}\frac{4}{J\pi }\int \phi {\Omega {}^{\prime }}^{\prime }\;\left[-\frac{1}{3}{Ī}_{4}I+J^{-2/3}f⊗f\right]⊗\left[-\frac{1}{3}{Ī}_{4}I+J^{-2/3}f⊗f\right]\;d\theta \\ & \;-\Phi_{\text{cf},t}\frac{4}{3J\pi }\int \phi \Omega^{\prime }\;(I⊗\left(-\frac{1}{3}{Ī}_{4}I+J^{-2/3}f⊗f\right)-\frac{1}{2}{Ī}_{4}(I_{\left(ikjl\right)}+I_{\left(iljk\right)})\\ & \;+J^{-2/3}(f⊗f)⊗I)\;d\theta . \end{array}$$
